# Nanoengineered chitosan functionalized titanium dioxide biohybrids for bacterial infections and cancer therapy

**DOI:** 10.1038/s41598-024-52847-1

**Published:** 2024-02-14

**Authors:** Mohammad Shabib Akhtar, Karthikeyan Chandrasekaran, Sharmila Saminathan, Siva Ranjani Rajalingam, Nehal Mohsin, Khalid Altigani Awad Alkarem Ahmed, Yasir Alhazmi, Ismail A. Walbi, Basel A. Abdel-Wahab, Amol D. Gholap, Md. Faiyazuddin, Gowri Sundaram

**Affiliations:** 1 Department of Clinical Pharmacy, College of Pharmacy, Najran University, Najran, 11001 Kingdom of Saudi Arabia; 2 Department of Chemical & Biochemical Engineering, Dongguk University, Seoul, Republic of Korea; 3 Department of Physics, Vel Tech Rangarajan Dr Sagunthala R&D Institute of Science and Technology, Chennai, India; 4 PG & Research Department of Physics, Cauvery College for Women, Tiruchchirappalli, Tamil Nadu India; 5 Department of Pharmacology, College of Pharmacy, Najran University, Najran, 11001 Kingdom of Saudi Arabia; 6 Department of Pharmaceutics, St. John Institute of Pharmacy and Research, Palghar, 401404 Maharashtra India; 7 School of Pharmacy, Al–Karim University, Katihar, Bihar India

**Keywords:** Biophysics, Cancer, Nanoscience and technology

## Abstract

Nanoengineered chitosan functionalized titanium dioxide biohybrids (CTiO_2_@NPs) were prepared with *Amomum subulatum Roxb* extract via one-pot green method and assessed by UV–Vis spectroscopy, XRD, SEM and EDAX analyses. As revealed by XRD pattern, the nanohybrids exhibits a rutile TiO_2_ crystallites around 45 nm in size. The emergence of the Ti–O–Ti bond is identified by observing a peak between 400 and 800 cm^−1^. A wide bandgap (4.8 eV) has been observed in CTiO_2_@NPs, due to the quantum confinement effects and the oxygen vacancies reveal the intriguing potential of developed nanohybrids for various applications. Surface flaws were identified by observing an emission band at 382, 437, 482, 517, and 556 nm. They also exhibit better antibacterial performances using well diffusion method against *Staphylococcus aureus*, *Bacillus substilis*, *Klebsiella pneumonia*, and* Escherichia coli*. CTiO_2_@NPs were discovered to have free radical scavenging activity on DPPH analysis and exhibit IC_50_ value as 95.80 μg/mL and standard (Vitamin C) IC_50_ is 87.62 μg/mL. CTiO_2_@NPs exhibited better anticancer properties against the osteosarcoma (MG-63) cell line. All these findings suggest that there is a forum for further useful therapeutic applications. Therefore, we claim that nano-engineered carbohydrated TiO_2_ phytohybrid is a promising solution for bacterial infections and bone cancer.

## Introduction

Green nanotechnology has emerged as a more environmentally friendly technology since it facilitates the synthesis of nanomaterials and nanoproducts with less energy, superior renewable inputs, and stronger biocompatibility and biodegradability features^1,2^. Nanotechnology has become a cutting-edge science with ground-breaking uses in various industry, including healthcare, textiles, food, the environment, and engineering. The increasing demand for nanotechnology in the pharmaceutical field is indeed a significant trend observed in recent years^3,4^. It is also used for biomedical actions including antimicrobial, anticancer, antioxidant, antifungal, etc. for effective biomolecular detection, drug delivery, waste treatment along with the food production^4–7^.

Osteosarcoma is a kind of bone cancer that primarily affects adolescents and young adults. By 2023, it is anticipated that 1000 people in America will be diagnosed with Osteosarcoma. Cancer can invade the brain if it gets recognized before in addition to spreading to other parts of the body^8–10^. The percentage of survivors who may continue to live for another five years is 76% for all age groups^8^. The primary causes of multidrug resistance in bacteria involve the development of genes that each code for resistance to specific agent on-resistance (R) plasmids or transposons, as well as the activation of multidrug efflux pumps, each of which may expel more than one drug^11^. There have been severe deteriorations in clinical conditions caused by drug-resistant bacteria^12–14^. To solve these problems and improve human health, various nanotechnological methods were employed^15^.

Nanoparticles can generally be produced using a physical, chemical, or biological technique. The first two approaches' use of hazardous chemicals to create nanoparticles will exacerbate the issue of chemical pollution and other issues with therapeutic applications^16^. Till date various metal oxide nanoparticles such as TiO_2_, ZnO, MgO, NiO, CuO, Co_3_O_4_, CeO_2_ and BaO being tailored biologically by using different natural extract (roots, seeds, flowers, fruits, leaves, barks, peel, algae, micro-organisms) to tailor its biological properties^4,6,16–25^. To overcome the environmental issues, preparing NPs via eco-friendly technique have a greater biocompatibility, non-toxicity, and cheaper manufacturing costs^23,26–32^. Many scientists have concentrated on the use of seeds as biomaterials in the assembly of nanoparticles. The bio-reduction of metal oxide nanoparticles from the extracts of seeds involved three stages: bioactivation, growth, and termination phase. Particle combinations through a spontaneous process engaged in the growth phase known as Ostwald ripening are present in certain metal ions as well as the nucleation process for reduced metal ions delivered into the activation phase. Several seed extracts were implemented as a reducing agent along with the stabilizing agent in the production of nanoparticles through seed constituents like flavones, ketones, sugars, amides, terpenoids, and carboxylic acids used for the bio-reduction process. These capping agents ensured about prevention of nanoparticle agglomeration and growth^33,34^.

Proteins present in the extract containing functionalized amino groups which participate in the reduction of the metal ions with the help of functional groups like –C–O–C, –C–O–, –C=C– as well as –C=O– for the same^5^. Phytocomponents, which are natural compounds derived from plants and seeds have the potential to serve as highly specific tools in cancer therapy. In comparison to microbial synthesis, bioreduction using seed extract has several advantages, including a higher rate of synthesis, an increase in monodispersity, effective stability for created nanoparticles, and a less involved process. The complexity of microbial synthesis is increased by the involvement of microbial separation, culture, and maintenance in the formation of nanoparticles. Based on the conceptual idea, some seed components, such as flavonoids, go through a tautomeric transformation from enol to keto form, and the latter form releases the hydrogen atom necessary for metal ion reduction for the creation of nanoparticles^5^. They can be engineered or designed to act as ligands, which are molecules that bind to certain receptors on the surface of cancer cells. The targeted approach enables treatments to be focused primarily on cancer cells while sparing healthy cells, thereby reducing collateral damage and side effects. By selectively targeting cancer cells, phytocomponent-based therapies have the potential to minimize damage to healthy tissues. This can lead to a significant reduction in treatment-related side effects, such as nausea, hair loss, and immune system suppression, which are commonly associated with traditional cancer treatments like chemotherapy and radiation therapy^5^.

One of the traditional seeds *Amomum subulatum* belonging to the Zingiberaceae family used as a curative and preventive drug for digestive disorders, lung congestion, and mouth infections owing to their essential oil constituents which majorly consist of 1,8-cineole followed by α-terpineol as well as as limonene etc. In addition, the seed has cancer prevention, anti-bacterial, and antioxidant properties that improve aroma^35,36^. Chitosan is a naturally derived product that can be applied in different areas due to its biocompatibility, biodegradability, and benign properties^37^. The chitosan induces the metal chelation to create a destabilized outside film of Gram-negative bacteria or that of the cell walls of microbes. The cell wall penetration of chitosan is facilitated owing to its electrostatic interaction with the cell surfaces with the help of an amino-protonated version of the same. Chitosan has shown great interaction with the microbial deoxyribose nucleic acid (DNA) for effective internalization of the same to interfere with the ongoing gene expression process^37^. It has shown antifungal action due to protonated chitosan interface with the negatively charged phospholipid components of fungi membrane. Such interactions triggered the enhancement of the permeability of the membrane resulting in cellular content leakage foremost to cell death^37,38^.

Titanium dioxide nanoparticles have been well-known for over a decade for their amazing contributions to the medical and agricultural fields. Due to their non-toxicity, inexpensive nature, impressive surfaces, non-corrosive, bio-compatibility and photo-catalytic properties, titanium dioxide nanoparticles (TiO_2_NPs) are one of the wide spread distributed particles for drug delivery, antibacterial materials, cosmetics, sunscreens, electronic industry^6,9^. Titanium dioxide nanoparticles (TNP) are used to alter the cellular mechanistic stress-dependent type of signaling pathways completed through Mitogen-activated protein kinase (MAPK) instigation followed by transcription factor activation for the same. The TNP can enter the cell through phagocytosis, pinocytosis, or that of micropinocytosis for get collected at specific locations in the cells^39^. The selection mechanism to enter the cells is based on the particle size of the developed nanoparticles while the incidence of the TNP in the cell will reorganize cells. In another study exposure of titanium dioxide nanoparticles to UV rays induces redox chemical reactions for the production of electrons which further strongly react with the nearby oxygen and water molecules to generate numerous reactive oxygen species like high levels of hydrogen peroxide, hydroxyl radicals, superoxide, and hydroperoxyl ions^2,40^. The peroxide and hydrogen peroxide moieties are sensitive to that of the oxidation carriers and are used to penetrate quickly into cells for influence on the subcellular organelles like nuclei or mitochondria etc. resulting in cancer cell death through disruption of cellular homeostasis. They also induce enhanced cell sensitization for a reduction in cell proliferation rate, organic specific carcinogenesis as well as mortality^41^.

The nanoformulation of titanium dioxide has demonstrated prevention of the cancer cell cycle to impact cell proliferation^42,43^. Since chitosan-decorated titanium dioxide nanoparticles (CTiO_2_@NPs) are semiconductor metal oxides, they exhibit excellent therapeutic properties when applied to various cancer cells through photodynamic, photothermal, sonodynamic, and near-infrared light therapy. As a result of their outstanding energy efficiency, thermal stability, activity as a photocatalyst, and low cost, they are used in cosmetics, food colorants, paints and inks, biosensors, and energy storage devices developed chitosan-titanium dioxide nanocomposite films with different concentrations^44–46^. One percent of TiO_2_-chitosan exhibits good ethylene degradation and antimicrobial properties against Gram-positive bacteria. According to earlier studies^26,47–49^ adding the capping agent has the effect of reducing the aggregation of prepared TiO_2_ NPs by strongly coordinating the protonated chitosan and Ti^2+^ ions on the surface of the developed TiO_2_ NPs.

In the present research, a green approach involving *A. subulatum* extract was used for synthesis for CTiO_2_@NPs as a bio-reducing agent from the precursor of titanium isopropoxide in addition to the inclusion of drug carriers as chitosan into the developed formulation. The purpose of adding a capping agent is to hinder the surface chemical reactivity of the developed CTiO_2_@NPs through strong coordination between the protonated chitosan and Ti^2+^ ions for the reduction of aggregation of prepared CTiO_2_@NPs^47^. Perhaps, we are reporting the use of flavonoids in synthesizing CTiO_2_@NPs for the first time in greener fusion of nanoparticles. The synthesis and application of nanomaterials can be done in a more sustainable way by implementing green nanotechnology. It seeks to reduce environmental impact, improve biocompatibility, and encourage the creation of novel solutions that support sustainable development objectives by fusing eco-friendly concepts with nanotechnology. This streamlines the procedure, which could cut down on resources and production time. When natural extracts are used, the synthesized nanomaterials' biocompatibility is generally improved, which is beneficial for biomedical applications including nano- engineering and medication delivery. Further, we present an inclusive range of characterization of developed CTiO_2_@NPs by utilizing XRD, FTIR, DLS, UV, PL, and SEM studies and biologically assessed their potential exploiting antibacterial, antioxidant, In vitro toxicity, and anticancer effects on human osteosarcoma cell lines.

## Results and discussion

### X-ray diffraction

**Figure 1:**
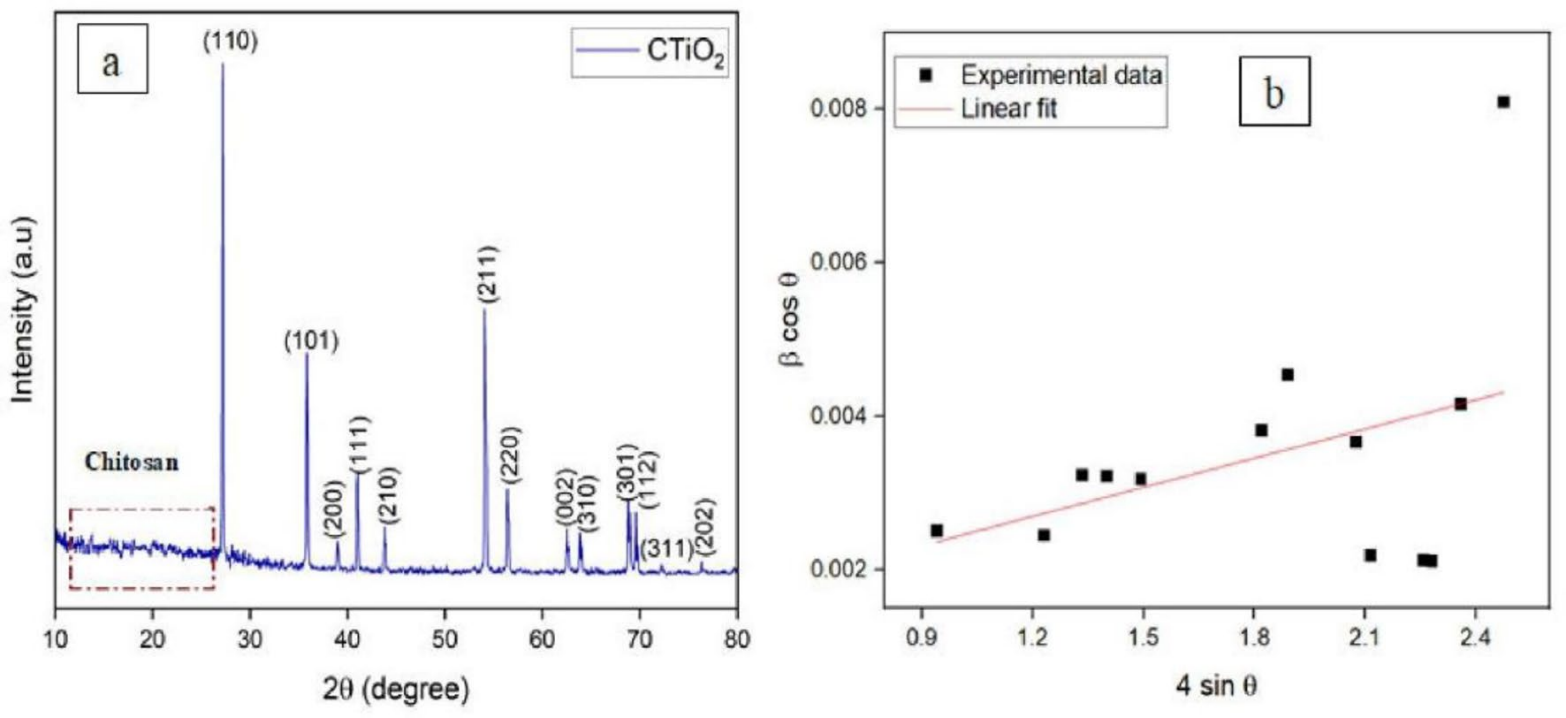
(**a**) X-ray diffraction pattern, and (**b**) W–H plot of CTiO_2_@NPs.

XRD pattern of bio-synthesized chitosan decorated TiO_2_ was exhibited in Fig. 1a. The pattern observed in the lower region of 2θ from 10 to 27° indicates the characteristic peak of chitosan^50,51^. The XRD peaks observed at 27.214, 35.855, 38.987, 41.019, 43.845, 54.1, 56.425, 62.495, 63.824, 68.771, 69.558, 72.267 and 76.454° correspond to (110), (101), (200), (111), (210), (211), (220), (002), (310), (301), (112), (311) and (202) planes respectively. The strong and highly crystalline diffraction peak observed from 27 to 80° indicates the formation of tetragonal structured rutile phase TiO_2_ with a *P42/mnm* space group. The obtained peaks agree with ICDD No. 21-1276 as well as with earlier reports^52,53^. The sharpness of the peak and lack of any other peaks indicated that only pure titanium dioxide nanoparticles had formed. These results showed the formation of nanoparticles through intermolecular hydrogen bonding between biomolecules and metal oxides^54^. These findings demonstrated the steric interactions between biomolecules and metal oxides that generate nanoparticles. This finding proves that the chitosan-based TiO_2_ nanomaterials surface matrix formed^54^. The average crystallite size of CTiO_2_@NPs was intended as 45 nm using the Debye–Scherer formula: D = 0.9λ/β cos θ, where D is the crystallite size, β represents the full-width half maximum, θ indicates Bragg’s angle and λ is the wavelength of X-ray. Figure 1b implicit the Williamson–Hall plot to examine the crystallite size and strain of the chitosan decorated TiO_2_ nanoparticles. The average crystallite size (48 nm) can be determined by the intercept of a linear fit to experimental measurements, and the lattice strain (0.00127 × 10^–4^) from the slope of the fitting. The development of a positive slope shows that the prepared material has naturally experienced tensile strain due to imperfections in the crystal lattice^55^.

### FTIR and UV spectroscopy

**Figure 2:**
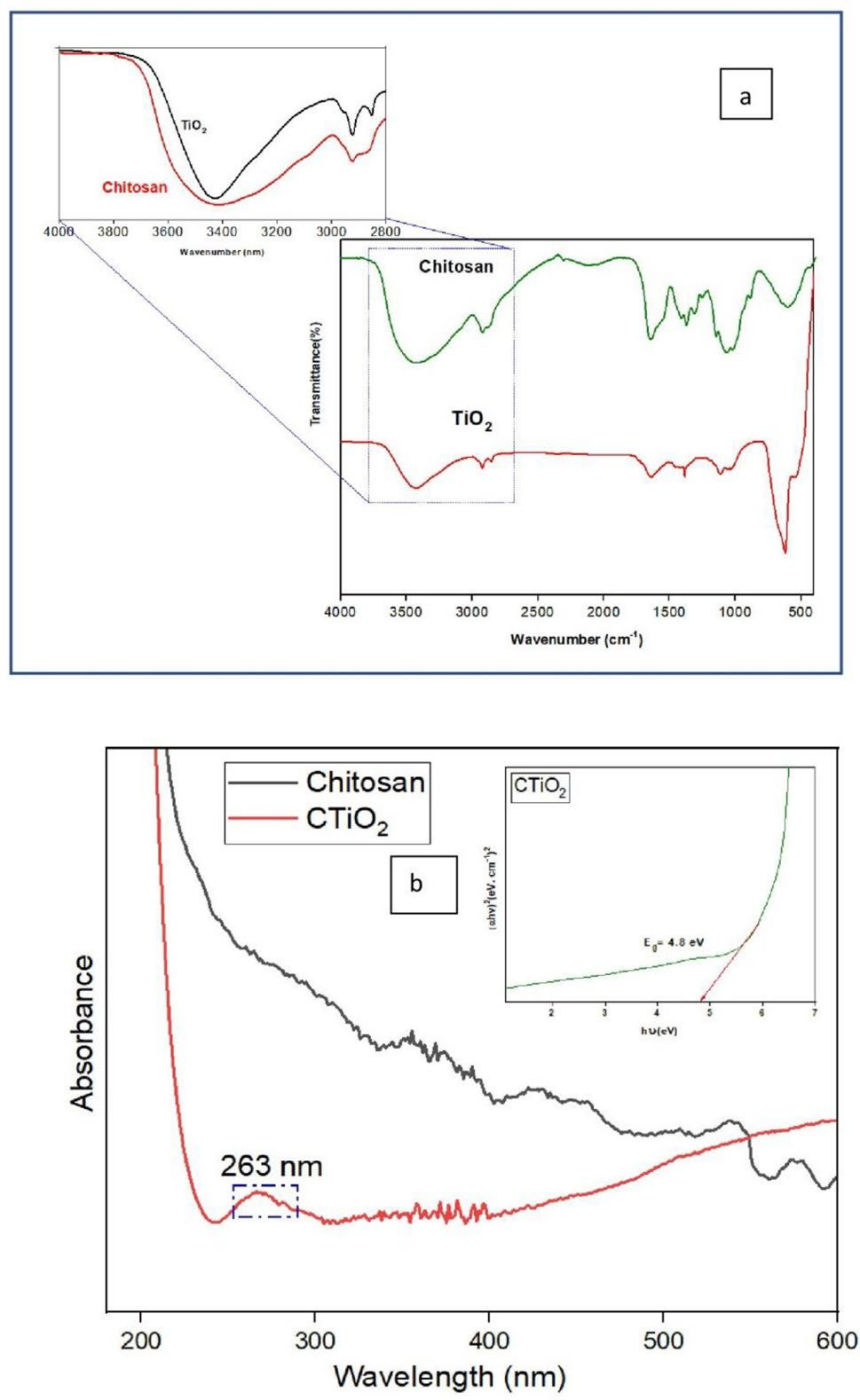
(**a**) FTIR spectrum, (**b**) UV–Vis. spectrum of CTiO_2_@NPs and Tauc plot (inset).

The various functional groups that are present and used in the formation of nanoparticles were identified from the FTIR spectrum, as revealed in Fig. 2a. The wide –OH and –NH peaks of chitosan with hydrogen bonds present near 3300 cm^−1^ and 1640 cm^−1^, demonstrating the amide group, which gets weaker in CTiO_2_@NPs. The weak peak centered at 2924 and 2852 cm^−1^ indicating the symmetric and asymmetric vibrations of –CH_3_ respectively. The characteristic peak at 1639 cm^−1^ is accompanied by the C=O stretching vibration of the acetamido groups of chitosan and the carboxylic acid group is confirmed by examining a peak at 1384 cm^−1^^8,43,56^. Many peaks observed from 1000 to 1500 cm^−1^ for chitosan are suppressed for CTiO_2_@NPs. The antisymmetric stretching of C–N and the stretching vibration of C–O–C are centred at 1113 cm^−1^ insist on hydrogen bonding between the –OH group of chitosan and Ti and the presence of polysaccharides in the chitosan molecule^57,58^. The band centered at 617 and 527 cm^−1^ is attributed to the bending vibration of Ti–O interaction with the surface of chitosan. The presence of various functional bioactive molecules was confirmed by FTIR data, which took part in converting Ti^2+^ into Ti^0^ during oxidation (on annealing). Later Ti converted into TiO_2_ NPs on green synthesis^56^. According to the findings, chitosan may establish a strong and high intermolecular hydrogen bond with TiO_2_.

Figure 2b displays the UV-absorbance spectra of pure chitosan and chitosan decorated TiO_2_ NPs. CTiO_2_ spectra exhibit a maximal absorbance at 263 nm due to the formation of nanoparticles, whereas pure chitosan does not exhibit any peak. This result is like the result obtained by^59^. The inset within Fig. 2b presents the calculated bandgap of the prepared NPs, which registers at 4.8 eV. This value exceeds the bandgap of bulk TiO_2_ (3.2 eV) and so many earlier reports which may be due to the quantum confinement, a blue shift occurs resulting in the broadening of the bandgap^59–61^. The surface plasmon resonance moved to the lower wavelength side due to the materials' increasing bandgap. These observations from the TiO_2_ NPs surface matrix may lead to oxygen vacancies, which would also enhance their biocidal qualities^56^.

### DLS analysis

**Figure 3:**
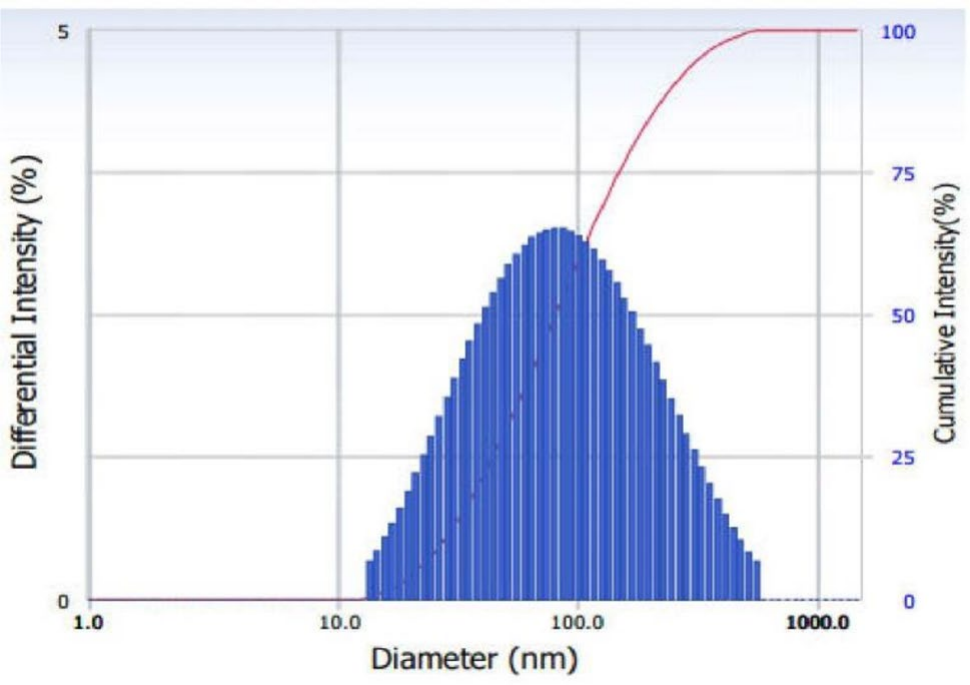
DLS spectrum of CTiO_2_@NPs.

The Dynamic Light Scattering (DLS) technique was utilized to study the dispersal of CTiO_2_@NPs as technique shown in Fig. 3. The TiO_2_ NPs hydrodynamic diameter and polydispersity index were calculated as 122 nm and 0.325. The hydrated chitosan that absorbs water molecules during DLS spectra analysis may be responsible for larger particle sizes than XRD measurements. It is attributed to the foreign impurities (chitosan) and the existence of several functional groupings in the extract which lead to distortion on the host TiO_2_ surface and are linked to electrostatic interaction or significantly larger intermolecular hydrogen bond formation in the chitosan-TiO_2_ matrix^57,62^.

### SEM analysis

**Figure 4:**
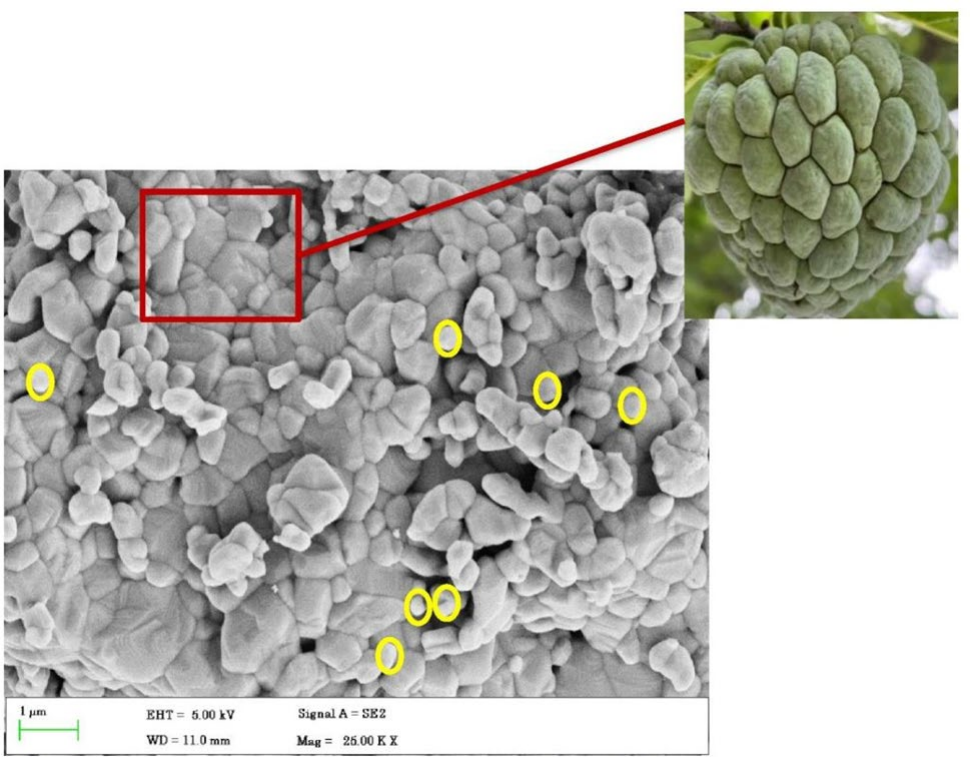
SEM image of CTiO_2_@NPs resembles the surface of custard apple.

SEM image of chitosan-decorated TiO_2_ is shown in Fig. 4. The results revealed a uniformly smooth surface with polygonal particles devoid of any agglomeration. The image resembles the surface of a custard apple. The presence of chitosan over titanium dioxide nanoparticles is implied by tiny spherical particles that are observable on the surface (yellow circle). The average particle size of CTiO_2_@NPs was observed at 60–80 nm.

### Photoluminescence analysis

**Figure 5:**
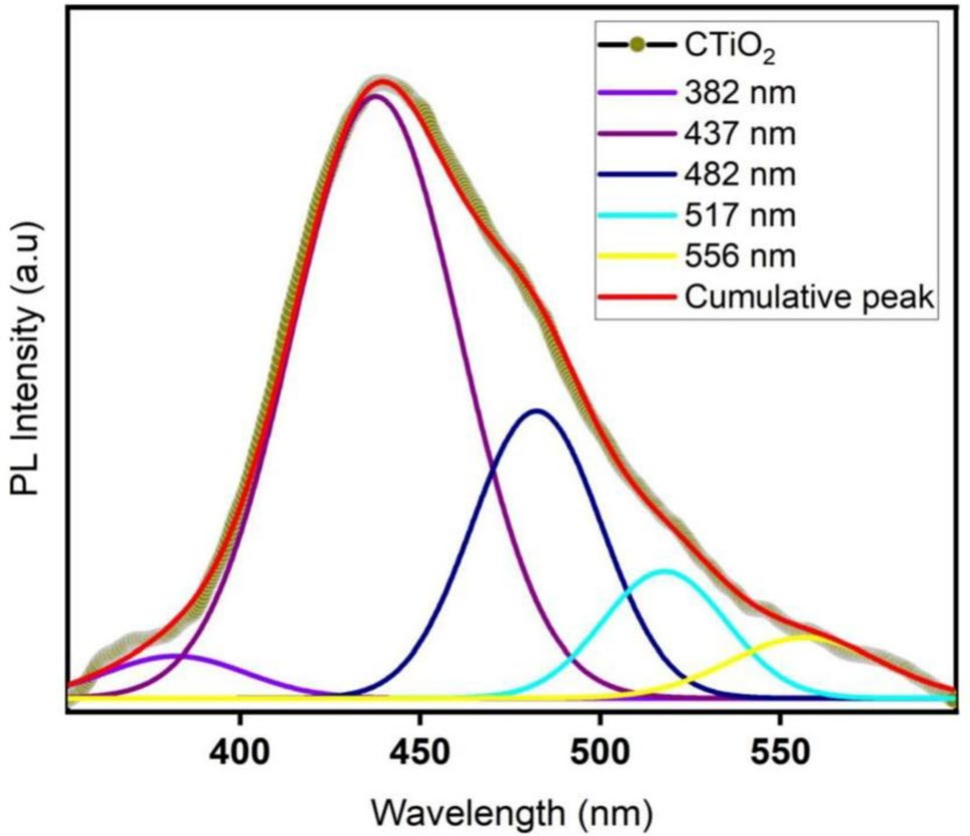
PL spectrum of CTiO_2_@NPs.

Using a light source that emits at a wavelength of 325 nm, the photoluminescence spectrum of CTiO_2_@NPs produced through the green method is depicted in Fig. 5. It will reveal any surface flaws and oxygen vacancies that exist in the prepared material. From the PL spectra, five emission peaks have been identified at 382, 437, 482, 517, and 556 nm. At 382 nm, near-band edge (UV emission) was detected due to exciton-exciton recombination processes; two blue emission peaks were identified at 437 and 482 nm because of oxygen vacancies and self-trapped Ti interstitials^54^. A photo-generated hole and an ionized electron in the valence band of TiO_2_ recombine, resulting in a green emission peak at 517 nm^35^. To produce free radicals, which play a part in biological performance, these oxygen vacancies and self-trapped Ti interstitials. In the PL of CTiO_2_@NPs, three types of physical origins have been detected in published samples, including oxygen vacancies, self-trapped excitons, and surface defects. The production of reactive oxygen species (ROS), which in turn damage the interior cytoplasmic membrane, is largely dependent on these surface defects. In addition, they have the ability to slowly expel proteins and DNA, which kills bacteria and cancer cells^56^.

### Antioxidant activity

**Figure 6:**
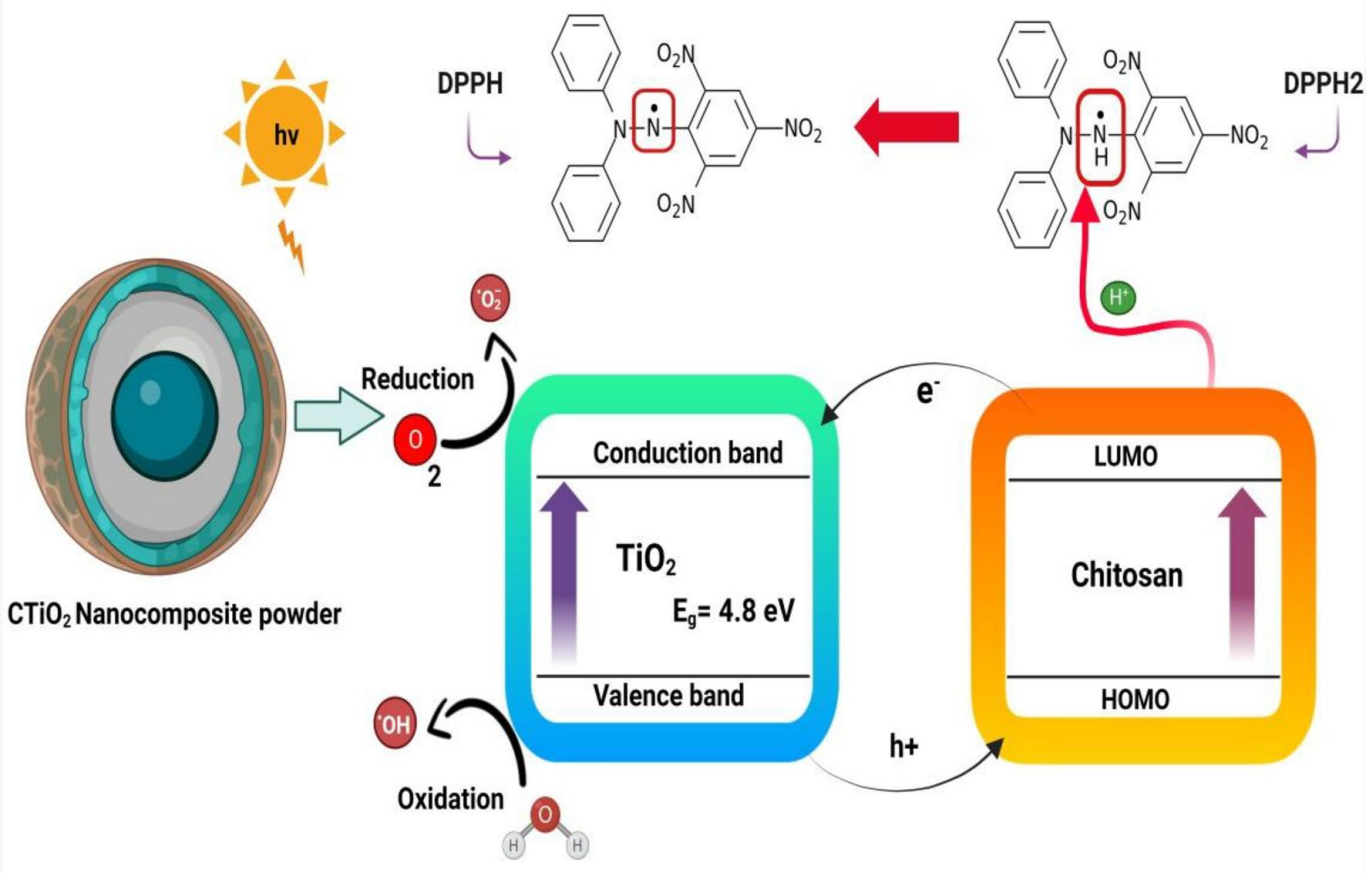
CTiO_2_@NPs used in antioxidant mechanisms of DPPH free radical scavenging activity when exposed to visible light.

**Figure 7:**
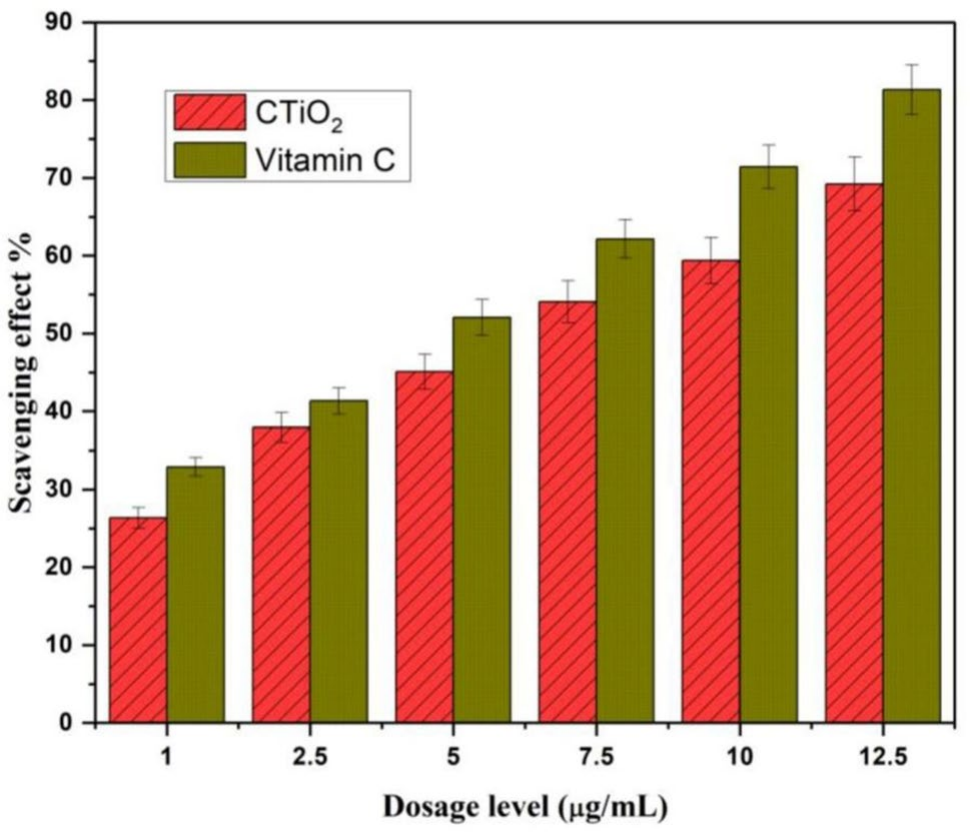
Antioxidant activity of CTiO_2_@NPs and vitamin C.

Upon reduction, the color shifts from purple to yellow, quantifiable a reduction in absorption at 517 nm. In this study, CTiO_2_@NPs displayed a dose-dependent capacity for scavenging radicals. The investigation revealed CTiO_2_@NP's efficacy in scavenging free radicals using DPPH. It involves a vital procedure that retains oxidative stress, membrane breakdown, and cell integrity under control. As depicted in Fig. 6, the radical scavenging effects enhance CTiO_2_'s light absorption capability, enabling activity against active free radicals. The antioxidant property of the prepared material is exhibited in Fig. 7. The sample exhibited a DPPH IC_50_ value of 95.80 μg/mL, while the standard (Vitamin C) IC_50_ was 87.62 μg/mL. The mixture's absorbance was measured spectrophotometrically at 517 nm.

### Antibacterial assay

The antibacterial properties of nanomaterials are heavily predisposed by their physicochemical attributes, including size, outline, surface chemistry, and configuration. These properties significantly affect the nanomaterial's interaction with microbial cells and can influence their efficacy in damaging cell membranes. The mechanisms through which nanomaterials exert antibacterial effects are complex and can involve various processes such as membrane disruption, activation of reactive oxygen species (ROS) and interference with cellular functions. Typically, the most prevalent antibacterial mechanism has been observed to involve the evolution of reactive oxygen species by nanoparticles. The key mechanism involved in antibacterial activity encompasses the rupture of the bacterial cell membrane, infiltration into the bacterial cell membrane, and initiation of intracellular antibacterial effects, which include interactions with DNA.

The pathogenic bacteria selected for this study can cause a wide range of infections, from minor skin infections to serious conditions including pneumonia and bloodstream infections. Some *E. coli* strains are also utilized in biotechnology and molecular biology for a variety of experimental objectives, including protein synthesis and genetic engineering investigations. *Klebsiella pneumoniae* is being studied by researchers to better understand its pathogenic processes, antibiotic resistance, and approaches to create novel therapies or preventative tactics against infections caused by these bacteria. *Bacillus subtilis*, on the other hand, is widely utilized as a model organism to study many aspects of bacterial biology and for industrial applications because to its safety and well-characterized genetics^63^.

**Figure 8:**
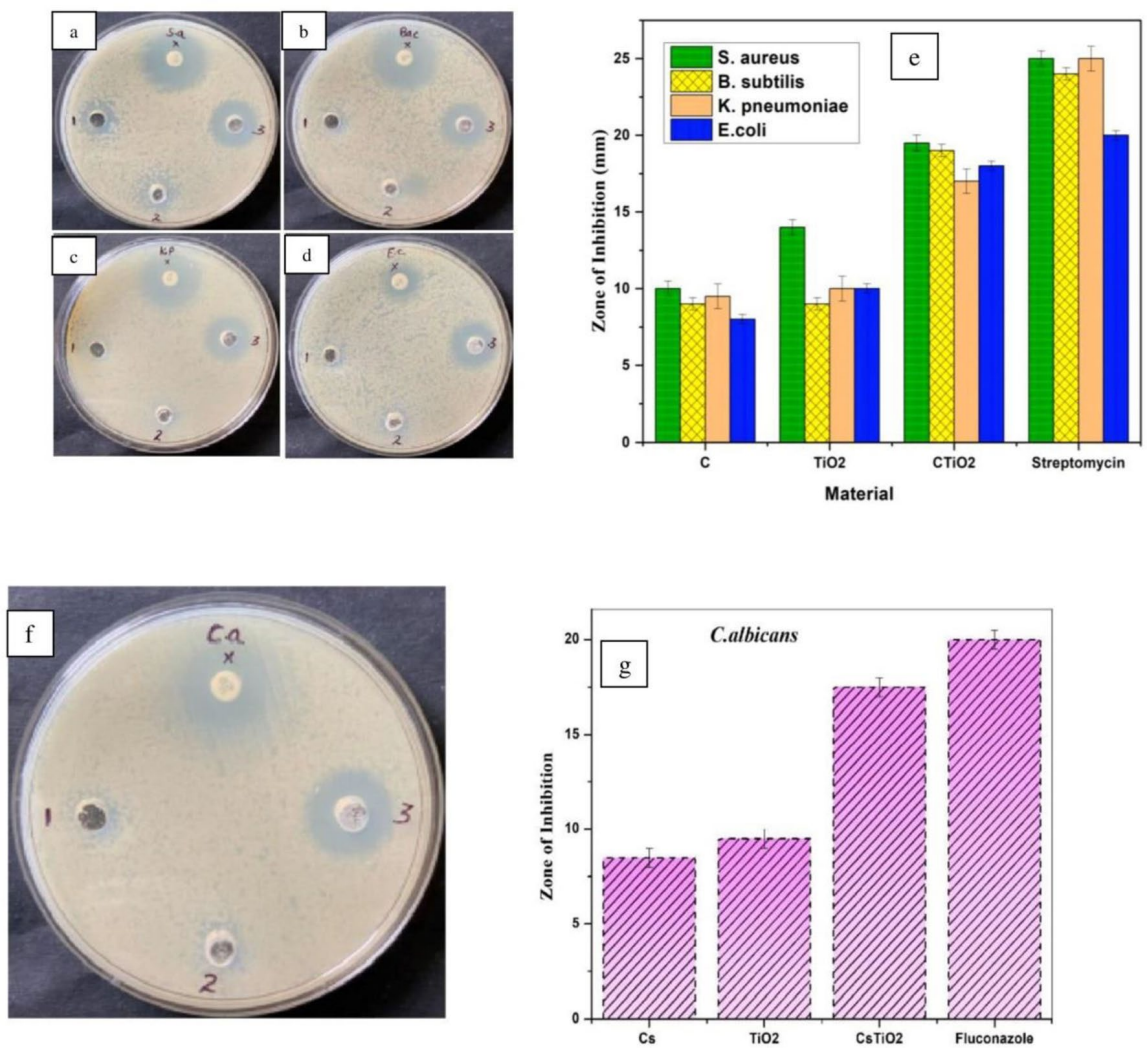
Antibacterial performance of CTiO_2_@NPs: (**a**–**e**) *Staphylococcus aureus*, *Bacillus substilis*, *Klebsiella pneumonia*, *Escherichia coli* and (**f**–**g**) Antifungal activity.

In this scenario, the produced nanoparticles was studied against *Staphylococcus aureus* (19.5 mm), *Bacillus substilis* (19 mm), *Klebsiella pneumoniae* (17 mm), and *Escherchia coli* (18 mm) as shown in Fig. 8a–d. From the results, the prepared CTiO_2_@NPs exhibits the most confined zone of inhibition against the cell wall. The antibacterial activity's extent is contingent on the material's concentration and the type of metal oxide employed, the cell wall of the bacteria is damaged by the prepared nanoparticles. The interaction between the nanoparticles penetrates the cell wall and causes its breakdown. Moreover, the electrons within the conduction band and the holes in the valence band display potent reducing and oxidizing properties, facilitating the generation of free radicals. These free radicals are recognized for their capacity to damage cells by disrupting DNA, amino acids, lipids, carbohydrates, and proteins. The antifungal activity of pure chitosan, TiO_2,_ and chitosan-TiO_2_ against the bacterium *Candida albicans is* shown in Fig. 8f–g. Due to their thick cell walls made of glucan and chitin, *C. albicans* yeast cells are more resistant to bacteria than other types of cells^64^. However the ethical considerations of metal oxide nanoparticles, especially in the biomedical context, are integral part of research in order to warrant their safety and efficacy. Table 1 highlights the antibacterial action of different bacteria taken in recent studies of TiO_2_ and present work.

**Table 1:** A landscape of antibacterial performance of CTiO_2_@NPs against different pathogens.

Plant species	Bacterial strain	Zone of inhibition (mm)	Bioactivity
*Trigonella foenum-graecum*	*Enterococcus faecalis*	11.4 ± 0.5	Antimicrobial^32^
	*Staphylococcus aureus*	11.2 ± 0.4	
	*Streptococcus faecalis*	11.6 ± 0.3	
	*Bacillus subtilis*	10.5 ± 0.4	
	*Yersinia enterocolitica*	10.6 ± 0.5	
	*Proteus vulgaris*	8.5 ± 0.4	
	*Escherichia coli*	10.8 ± 0.3	
	*Pseudomonas aeruginosa*	10.3 ± 0.4	
	*Klebsiella pneumoniae*	10.2 ± 0.8	
*Mentha arvensis*	*Proteus vulgaris*	15	Antibacterial, antifungal^65^
*Psidium guajava*	*Staphylococcus aureus*	25	Antibacterial, antioxidant^66^
	*Escherichia coli*	23	
*Azadirachta indica*	*Staphylococcus aureus*	20.83	Antibacterial^67^
	*K. pnuemoniae*	16.66	
	*B. subtilis*	25	
	*S. typhi*	10.42	
	*Escherichia coli*	10.42	
*Acorus calamus*	*Escherichia coli*	6 ± 0.3	Antimicrobial^68^
	*Pseudomonas aeruginosa*	8 ± 0.6	
	*Bacillus subtilis*	8 ± 0.4	
	*Staphylococcus aureus*	11 ± 0.6	
*Amomum subulatum*	*Staphylococcus aureus*	19.5 ± 0.5	Antibacterial, anticancer, antioxidant, antifungal (present work)
	*Bacillus subtilis*	19 ± 0.4	
	*Klebsiella pneumoniae*	17 ± 0.8	
	*Escherichia coli*	18 ± 0.3	
	*Candida albicans*	10.2 ± 0.5	

### Anticancer effect

**Figure 9:**
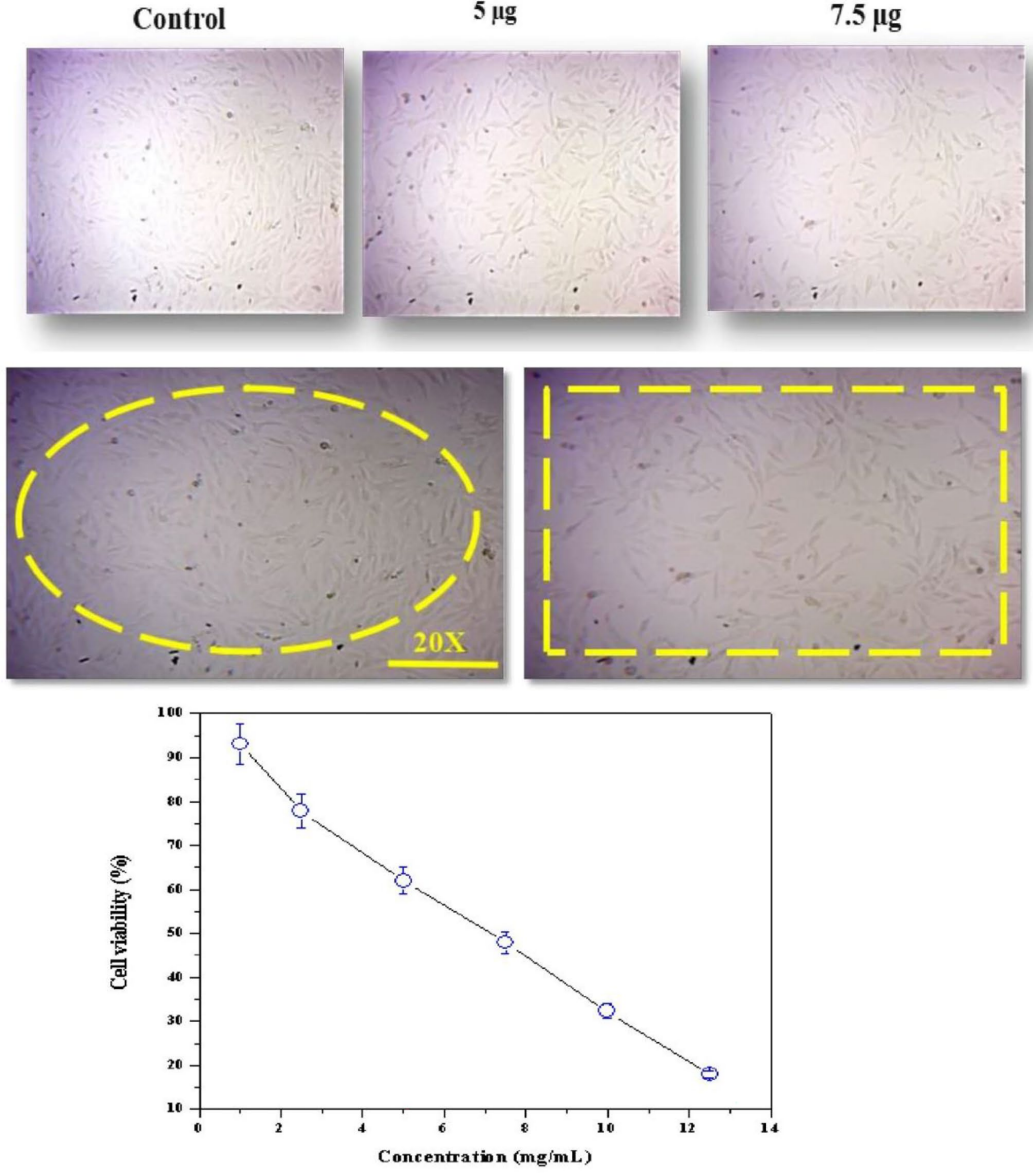
Morphological changes in MG-63 cells and Toxicity studies of MG-63 cells treated with various (10–100 µg/mL) concentrations of CTiO_2_@NPs for 24 h.

**Figure 10:**
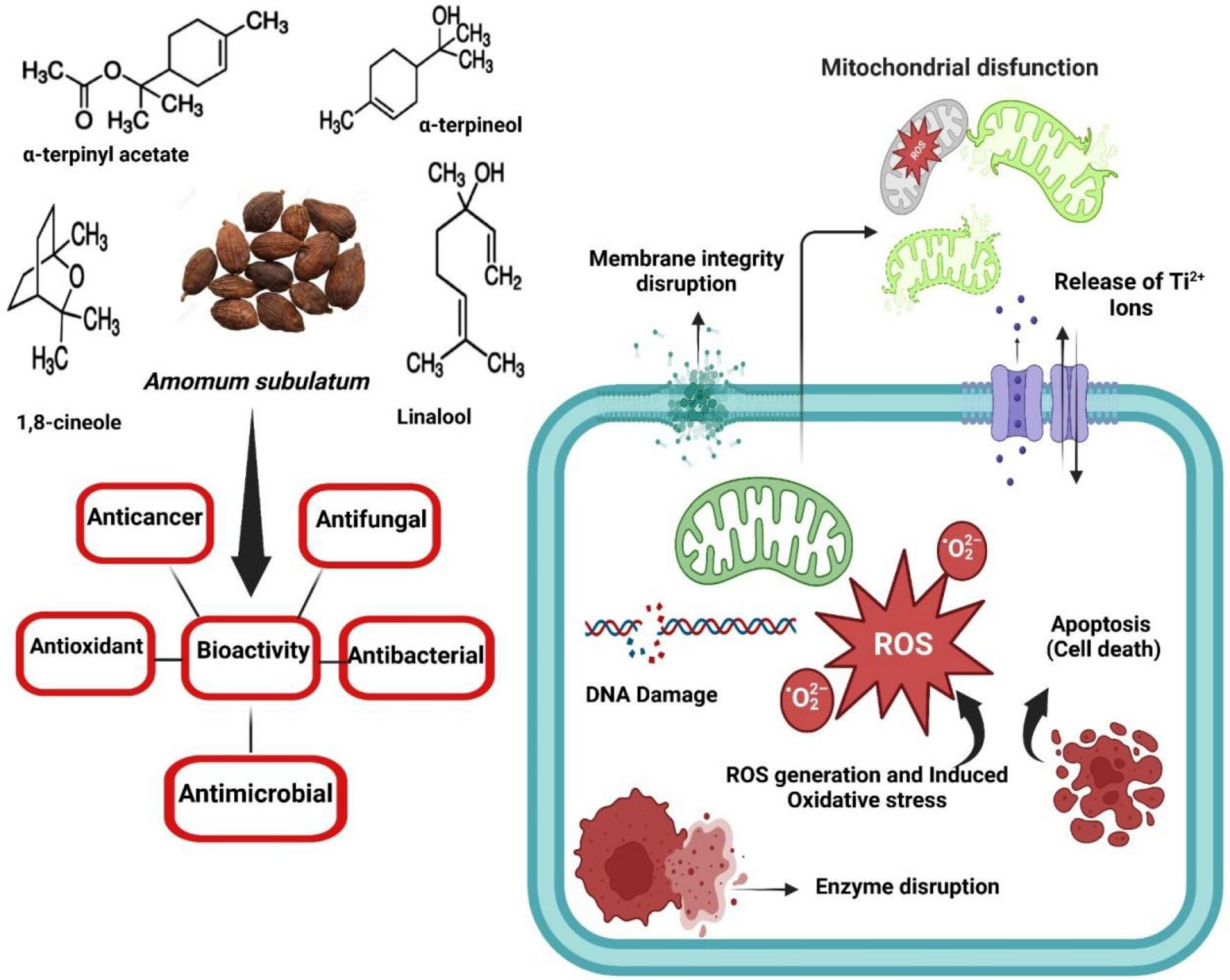
Schematic representation of the possible mechanism involved in *A. subulatum* and CTiO_2_@NPs in anticancer activity.

For anticancer investigation, different concentrations of CTiO_2_ hybrid nanomaterials were tested for MG-63 cell lines, as presented in Fig. 9. When MG-63 cells are treated with CTiO_2_ (5 and 7.5 g/mL for 24 h), compared to the control group, the photomicrograph (20x) presented in Fig. 9 illustrates the cellular morphological alterations, including shrinkage, detachment, membrane blebbing, and distorted shape^69^. A consistent fusiform shape can be seen in the untreated control cell (93% cell viability) as presented in Fig. 9. Hybrid nanomaterials in MG-63 cells were significant (p ≤ 0.05). Results obtained for CTiO_2_ a hybrid nanomaterial was contrasted with earlier TiO_2_ research on the cytotoxic response between different cell types. As highlighted below, the potential mechanisms underlying cancer cell death encompass various aspects. Figure 10 illustrates how CTiO_2_@NPs aid in the demise of eukaryotic cells by playing a role in ROS. This mechanism involves: (a) Generation of active free radicals like singlet O_2_ and OH radicals through water splitting within cells. This oxidative stress can damage bacterial cell walls, particularly the peptidoglycan layer and inner cytoplasmic membrane. (b) These effects lead to disturbances in respiratory functions, involving gradual RNA and protein leakage alongside rapid K^+^ ion leakage. Protein adsorption, rate of dissolution, ROS production, and the liberation of Ti^2+^ ions all have an impact on a nanomaterial's toxicity. The cytotoxicity percentage of TiO_2_ NPs is significantly governed by the elevated levels of ROS generated, intensifying their impact. Consequently, CTiO_2_@NPs exhibit noteworthy anticancer activity, particularly against osteosarcoma cells. When compared to other metal oxides from earlier investigations utilizing different cell lines, CTiO_2_ cytotoxic response was found to be superior (Table 2). It might be caused by the presence of chitosan, which interacts with molecules of TiO_2_ to form a matrix. However, TiO_2_ a very potential nanomaterial will be appropriate for pioneering therapeutic applications in the medical sectors. The physicochemical activity of nanoparticles is enhanced by their small size, which ends up in an elevated surface-volume ratio. Numerous surface flaws are crucial for the biocidal capabilities of nanoparticles since they have a substantial impact on cytotoxicity. Intracellular oxidative stress may be brought on by metal oxide nanoparticles entering the cell by a potential route^56^.

**Table 2:** Cytotoxic response of metal oxide over various cell lines.

Materials	IC_50_ concentration	Cell lines	Reference
ZnO, CZnO & AZnO	32 μg/mL 64 μg/mL 16 μg/mL	Hep-G2-Hepatic cancer	^25^
MgO & CMgO	800 & 1000 μg/mL 500 & 800 μg/mL	MCF-7- Breast cancer	^50^
Ag-MgO	15 mm 14 mm	A549-cancer cells (adeno carcinomic human alveolar basal epithelial Cells)	^31^
ZnO & TiO_2_	12.59 μg/mL	Human breast cancer (MDA-MB-231) and fibroblast (L929) cell lines	^54^
TiO_2_ & ZnO	20–100 μg/mL	MCF7-Breast cancer	^26^
Chitosan decorated TiO_2_	6.5 μg/mL	MG-63-Osteosarcoma cell line	(Present work)

## Material and methods

### Chemical and reagents

Titanium(IV) isopropoxide (MW: 284.21532 g/mol), chitosan (MW: 80–120 kDa), acetic acid (≥ 99.7%), acetic acid (98%) and ethanol (≥ 99.5%) were obtained from Sigma Aldrich chemicals. *A. subulatum* (Black cardamom seeds) were purchased from Goodness grocery—Amazon. Chemicals used were purchased in analytical grade and were obtained from Sigma-Aldrich. Throughout the procedure, double-distilled water was employed.

### Preparation of natural reducing agent and titanium oxide nanoparticles

Dried *A. subulatum* powder (5 g) is added to 100 mL of ethanol to prepare the homogenous solution under constant swirling and heating at 80 °C for 20 min. Filtered with Whatman No. 1 filter paper, the mixture was then stored in cold storage, which will serve as a reducing agent to prepare chitosan-decorated titanium dioxide nanoparticles.

TiO_2_ nanoparticles will be produced by dissolving 0.1 M of titanium (IV) isopropoxide in 80 mL of double-distilled water to produce an aqueous solution. Subsequently 20 mL of *A. subulatum* extract was incorporated into the homogenous solution, yielding a combination of milky white and yellow hues. The resulting solution was continuously stirred.

### Green synthesis of cross-linked chitosan passivated TiO_2_ nanohybrids

**Figure 11:**
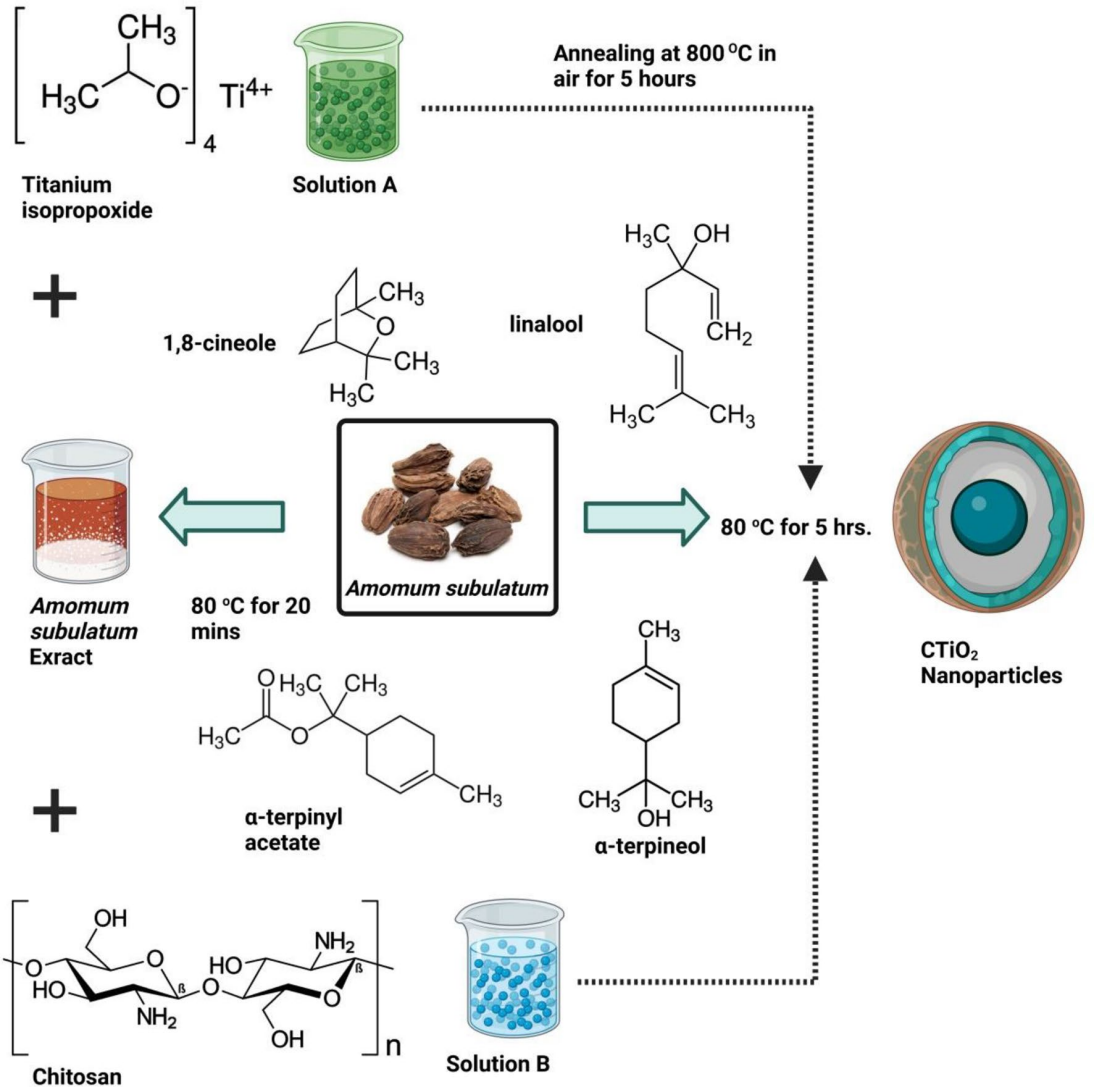
Schematic depiction of CTiO_2_@NPs.

To make a chitosan solution, dissolve 1 g of the powder in 100 mL of a 1% acetic acid solution. The homogeneous solution of chitosan is blended with the titanium metal solution while continuously stirring at 80 °C for three hours to create chitosan-passivated TiO_2_ NPs. After drying at 100 °C for two hours the ash white mixture was obtained. The resulting powder is then used for further study CTiO_2_@NPs after being annealed in air for 5 h at 300 °C. An illustration of CTiO_2_@NPs is revealed in the Schematic representation Fig. 11.

### Ethical approval

The national guidelines were followed in the collection and usage of the natural resources. There is no specific permit required to use this species for experimental purposes. Every technique employed in this research adheres with existing institutional, governmental, and worldwide regulations. *Amomum subulatum Roxb*. is not categorized as a threatened species or on the Red list, according to our check of the IUCN database.

### Characterization

#### X-ray diffraction

To analyze the formation and crystallite size of the produced material CTiO_2_, an X-ray diffractometer, X'Pert PRO (MODEL: X' PERT PRO—PANalytical) is also used for texture analysis, which involves studying the orientation and distribution of crystalline grains within a given material and recognized for its high precision and accuracy. The diffraction patterns in the bottom region of the *2*θ range fitted with CuKα radiation (λ = 0.1540 nm) ranges between 10 and 80° with a step size of 0.0500° was equipped to analyze the structure and materials prepared with different crystallite size.

#### FTIR and UV spectroscopy

Fourier Transform-Infrared Spectroscopy PERKIN ELMER model with spectrum two method was applied for the functional group determination present in the CTiO_2_ range from 4000 to 400 cm^−1^. Due to its advanced technology and software capabilities, using the PERKIN ELMER instrument to identify organic and inorganic materials precisely gives high-quality, dependable findings. However, UV–VIS spectroscopy analysis was performed using (JASCO V 750) to calculate the optical properties and band gaps of the material in the range of between 200 and 1100 nm. The materials or compounds are examined based on how they react to ultraviolet light, fall in the region between 180 and 380 nm, and visible region fall between 380 and 750 nm wavelength range.

#### DLS analysis

The UV–Vis Dynamic light scattering (DLS) using Nano Plus model spectrum was acquired to estimate the particle size distribution. The apparatus can measure the particle size of materials suspended in liquids ranging from 0.6 nm to 10 m at concentrations ranging from 0.00001 to 40%. To prepare the representative solution, the powdered CTiO_2_@NPs were thoroughly dissolved in HCl and then ultrasonically distributed to ensure a uniform distribution of NPs.

#### SEM analysis

Scanning Electron Microscopy (SEM) is employed for characterizing the surface morphology, composition, and microstructure of specimens including very high water vapor pressure of up to 3000 Pa. The surface morphology and the material's size were investigated by Scanning Electron Microscopy (SEM) using a JEOL JSM 6390 model. SEM provides high-resolution imaging of the surface of a specimen.

#### Photoluminescence study

The Perkin Elmer-LS 55 spectrometer was employed to obtain photoluminescence (PL) spectra. It gives useful information on the electrical and optical characteristics of materials. It has monochromators on both the excitation and emission sides, allowing it to do excitation (200–800 nm) and emission (200–900 nm) scans.

#### Antioxidant activity

A 0.1 mL solution of DPPH in methanol (0.135 mM) was added to 1.0 mL of different dosages of CTiO_2_@NPs. The reaction mixture was thoroughly centrifuged before being placed in a dark environment at ambient temperature for 30 min. The absorbance of the mixture was measured spectrophotometrically at 517 nm. Vitamin C was employed as a routine medication^30^. The proportion of neutralizing free radicals was used to compute using the equation below:

$$ \% {\text{ Scavenging }} = { 1}00 \, {-} \, \left( {{\text{A}}_{{\text{b}}} {\text{sample }}{-}{\text{ A}}_{{\text{b}}} {\text{blank}}} \right)/{\text{A}}_{{\text{b}}} {\text{control }} \times { 1}00. $$

(Where A_b_ represents absorbance).

#### Antibacterial assay

Following the Clinical Laboratory Standards Institute (CLSI), the antibacterial performance of the synthesized CTiO_2_@NPs was validated against pathogens. The bacteria were cultivated on nutrient agar to acquire the strain. 100 mL of a new culture containing 1 × 10^8^ CFU mL^−1^ of bacteria was disseminated onto Mueller Hinton Agar (MHA) plates with a sterile swab. To evaluate the bacterial stain, sterile filter paper of 6 mm diameter was placed on the outer surface of the contaminated agar plate and incubated for 24 h at 37 °C with three distinct concentrations of samples (1, 1.5, and 2 µg mL^−1^). Streptomycin was used as a positive control, with DMSO as a negative control. The biological activity of the prepared CTiO_2_@NPs was tested against two Gram-negative bacteria: *Escherichia coli* (MTCC 732), *Klebsiella pneumoniae* (MTCC 741), and two Gram-positive bacteria: *Bacillus subtilis* (MTCC 441), *Staphylococcus aureus* (MTCC 3160)^70^. The disc diffusion technique was used and alleviated to study the antibacterial activity of TiO_2_ nanoparticles. Using 30 mL of nutrient agar medium the petri plates were prepared. To obtain the bacteria’s strain, it was dispersed across nutrient agar. To investigate the bacterial strain, the sterile filter paper containing samples of 50 L (50 µg mL^−1^), 100 L (100 µg mL^−1^), and 150 L (150 µg mL^−1^) was distributed on the surface on the infected agar plate and incubated for 24 h at 37 °C in aerobic conditions. Chloramphenicol was used as a reference. The procedure was performed three times. The inhibitory zone that developed around the disc was measured in millimeters.

#### In vitro toxicity assay

In the current study, MG-63 cells were kept in growth media made up of Dulbecco's Modified Eagle Media (DMEM), by incorporating an added product of 10% foetal bovine serum (FBS), Penicillin (100 U/mL and streptomycin (100 μg/mL) was added to the medium to prevent bacterial contamination. Osteosarcoma (MG-63) cell lines were collected from the Cell repository of the National Centre for the National Centre Sciences (NCCS), Pune, India. A humid atmosphere was kept around the medium containing the cell lines with 5% CO_2_ at 37 °C^54^.

#### Scavenging activity of 2, 2-diphenyl-1-picrylhydrazyl (DPPH) radicals

The effect of CTiO_2_ on DPPH radicals was estimated using the method of^71,72^ 1.0 mL of various concentrations of CTiO_2_ was mixed with 0.1 mL of DPPH-methanol solution (0.135 mM). After proper vortexing, the reaction mixture was allowed to cool at room temperature for 30 min. At a wavelength of 517 nm, the absorbance by the mixture was determined through a spectrophotometer. Vitamin C was used as a standard drug. The percentage of free radical scavenging activity was calculated using the following equation:

$$ \% {\text{ Scavenging }} = { 1}00 \, {-} \, \left( {{\text{Abs sample }}{-}{\text{ Abs blank}}} \right)/{\text{Abs Control }} \times { 1}00. $$

#### Anticancer study

Cell viability assay, MG-63 cells were removed tallied, and counted using a hemacytometer. They were seeded in 96-well plates at a density of 1 × 10^4^ cells/mL and incubated for 24 h to consent adhesion. Following the application of various concentrations of TiO_2_ (1 to 12.5 g/mL) to each well, MG-63 cells were dealt with as the control. For 24 h, MG-63 cells were stored in a humid and warm environment containing a blend of 95% oxygen and 5% carbon dioxide. The MTT (5 mg/mL in PBS) dye was applied to each well after the drug-containing cells had been cultured for the first 4 h at 37 °C, and they were then washed with fresh culture media. A small volume of 100 µL of concentrated DMSO was used to dissolve with purple precipitate formation observed and the cell viability was measured by absorbance at 540 nm using a multi-well plate reader. The proportion of stable cells related to the control was used to represent the results. The appropriate dosages were investigated at various time points and the half maximum inhibitory concentration (IC_50_) values were derived. The IC_50_ values were calculated based on the TiO_2_ dose–response curve, which showed 50% less cytotoxicity than control cells. To achieve accurate findings, each experiment was carried out in duplicate at least three times^54^.

### Statistical analysis

Statistical analysis was conducted using one-way ANOVA with Tukey's multiple comparisons test and a significance level of P 0.05 (95% confidence interval). Graph-Pad Prism Software-VI was used for all statistical analyses, which were performed in triplicate.

## Conclusion

The current study provides an in-depth evaluation of the biological performance of bioactive CTiO_2_@NPs by leveraging the feasibility of an extract obtained from *A. subulatum Roxb.* The XRD patterns exposed that green synthesized CTiO_2_@NPs unveiled a tetragonal rutile structure with a *P42/mnm* space group. Furthermore, FTIR analysis unveiled the presence of intermolecular hydrogen bond formations between chitosan and TiO_2_ nanoparticles play a pivotal part in attracting the stability of the nanoparticles. Based on UV research, CTiO_2_@NPs has a comparatively large bandgap of 4.8 eV due to the quantum confinement effect. Through SEM analysis, the resultant nanoparticles take on a surface structure of custard apples with clear grain boundaries. Oxygen vacancies and self-trapped Ti interstitials from PL experiments are utilized to gauge its efficiency. CTiO_2_@NPs and amoxicillin samples exhibited antibacterial activities against G+ and G− bacteria with increasing the concentration of NPs increased their antibacterial activity. We also explored the antioxidant capabilities of CTiO_2_@NPs by subjecting them to DPPH radicals that outperformed the scavenging activity. The resultant CTiO_2_@NPs exhibited more anticancer activity at 6.5 μg/mL with minimum inhibition concentration of exciting possibilities against bone cancer (MG-63) cells. Overall, the produced NPs show great potential for usage in biomedical sectors with numerous applications (antibacterial and anti-cancer medications) in the future. Future research might concentrate on setting medicines into these nanohybrids and assessing their controlled release patterns. Furthermore, intensive research of green nanotechnology toward theranostics applications in personalized medical technologies is essential to ensure their commercial applications.

## Data Availability

All analysis during this study is included in this article as its supplementary information files.

## Abbreviations

*A. **subulatum*: *Amomum subulatum* Roxb.
TiO_2_: Titanium dioxide
CTiO_2_@NPs: Chitosan decorated titanium dioxide
NPs: Nanoparticles
ROS: Reactive oxygen species
DPPH: 2,2-Diphenylpicrylhydrazyl
IC_50_: Half-maximum inhibitory concentration
SEM: Scanning electron microscopy
DLS: Dynamic light scattering
Ti: Titanium
